# “*Escalibur*”—A practical pipeline for the de novo analysis of nucleotide variation in nonmodel eukaryotes

**DOI:** 10.1111/1755-0998.13600

**Published:** 2022-03-02

**Authors:** Pasi K. Korhonen, Babak Shaban, Noel G. Faux, Liina Kinkar, Bill C. H. Chang, Daxi Wang, Bicheng Yang, Neil D. Young, Robin B. Gasser

**Affiliations:** ^1^ 2281 Department of Veterinary Biosciences Melbourne Veterinary School The University of Melbourne Parkville Victoria Australia; ^2^ 2281 Melbourne Data Analytics Platform (MDAP) The University of Melbourne Parkville Victoria Australia; ^3^ 213636 BGI‐Shenzhen Shenzhen China; ^4^ BGI Australia, Oceania BGI Group Herston Queensland Australia

**Keywords:** bioinfomatics/workflows, molecular evolution, parasitology, population genetics

## Abstract

The revolution in genomics has enabled large‐scale population genetic investigations of a wide range of organisms, but there has been a relatively limited focus on improving analytical pipelines. To efficiently analyse large data sets, highly integrated and automated software pipelines, which are easy to use, efficient, reliable, reproducible and run in multiple computational environments, are required. A number of software workflows have been developed to handle and process such data sets for population genetic analyses, but effective, specialized pipelines for genetic and statistical analyses of nonmodel organisms are lacking. For most species, resources for variomes (sets of genetic variations found in populations of species) are not available, and/or genome assemblies are often incomplete and fragmented, complicating the selection of the most suitable reference genome when multiple assemblies are available. Additionally, the biological samples used often contain extraneous DNA from sources other than the species under investigation (e.g., microbial contamination), which needs to be removed prior to genetic analyses. For these reasons, we established a new pipeline, called *Escalibur*, which includes: functionalities, such as data trimming and mapping; selection of a suitable reference genome; removal of contaminating read data; recalibration of base calls; and variant‐calling. *Escalibur* uses a proven gatk variant caller and workflow description language (WDL), and is, therefore, a highly efficient and scalable pipeline for the genome‐wide identification of nucleotide variation in eukaryotes. This pipeline is available at https://gitlab.unimelb.edu.au/bioscience/escalibur (version 0.3‐beta) and is essentially applicable to any prokaryote or eukaryote.

## 1 INTRODUCTION

The use of advanced sequencing technologies has led to a revolution in genomics and an explosion in the number of genome‐wide sequence data sets for a broad range of organisms. The “democratization” of these technologies (Goodwin et al., 2016) has meant that the focus has extended from model organisms, such as *Caenorhabditis elegans* (C. elegans Sequencing Consortium, 1998) and *Drosophila melanogaster* (Adams et al., 2000) to nonmodel organisms (both invertebrates and vertebrates). For example, there has been a major expansion in genomic and associated proteomic and transcriptomic data sets for socioeconomically important parasitic worms of animals and plants (Kikuchi et al., 2017; Ma et al., 2020; Wit & Gilleard, 2017). This major and rapid progress has meant that the focus is now much more on analyses of massive genome data sets, which demand novel, efficient, reliable and reproducible tools as well as solutions to deal with this “data overload". Although a number of software workflows, such as cloudmap (Minevich et al., 2012), churchill (Kelly et al., 2015), toggle (Monat et al., 2015) and sarek (Garcia et al., 2020), have been developed to handle and process such large data sets and to carry out biological and/or population genetic analyses of them, there has been somewhat of a delay in the creation of effective, specialized workflows and pipelines for genetic and statistical analyses.

Most available workflows use the Genome Analysis Toolkit (gatk) (McKenna et al., 2010) for the identification and recording of nucleotide variation (i.e., “variant calling”). Commonly, the quantification of genetic variation within well‐known and well‐studied species relies on the use of one or more high‐quality reference genomes (NCBI) and variome data sets (e.g., for humans or mice; https://www.humanvariomeproject.org/) available in public or specialized databases. However, for most species, such extensive resources are not available. Frequently, an incomplete or fragmented genome is available, and sometimes little or nothing is known about a taxon's species‐status, its ploidy, whether sibling species exist, and/or whether genomic rearrangements occur within or among populations. Depending on the method used to collect samples, there may also be challenges linked to contamination with extraneous DNAs (e.g., viruses, bacteria and fungi). Thus, for ab initio studies of genetic variation in “lesser studied” eukaryotes, there is a need to ensure that well‐defined steps are put in place for the selection of suitable reference sequences (draft or reference quality) and that extraneous/nonspecific (“contaminating”) DNA sequences are removed prior to analysis.

For these reasons, we developed here a highly efficient, scalable pipeline for the genome‐wide analysis of nucleotide variation in eukaryotes. This pipeline, called *Escalibur*, has key steps, including nucleotide sequence trimming and mapping; selection of an optimum reference genome; removal of contaminating sequences; calibration; and variant calling. *Escalibur* version 0.3‐beta (available at https://gitlab.unimelb.edu.au/bioscience/escalibur) is suitable for the analysis of genetic variation among hundreds to thousands of individuals or samples of a eukaryotic species.

## 2 MATERIALS AND METHODS

**FIGURE 1:**
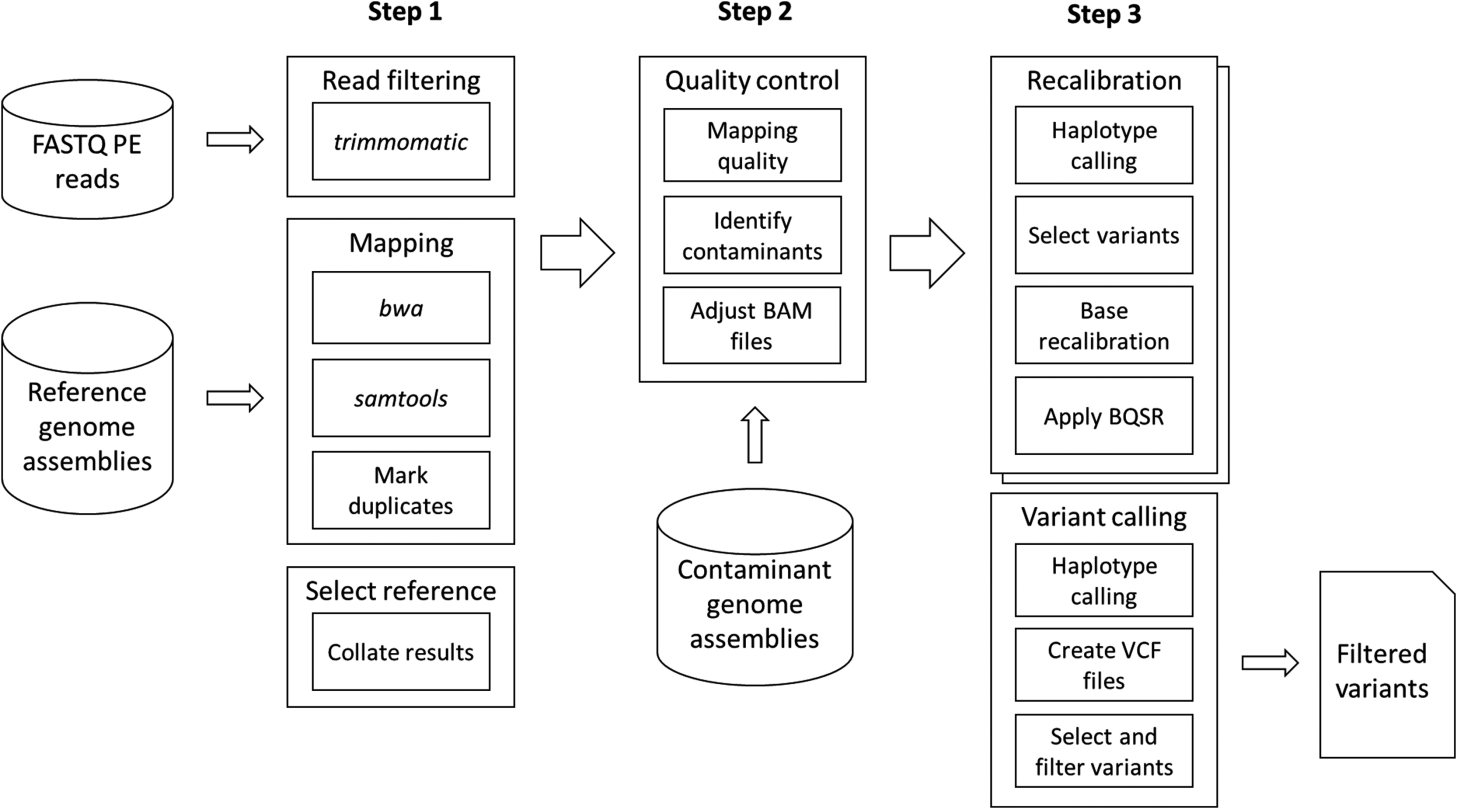
Schematic representation of the genomic analysis pipeline, *Escalibur*. In step 1, paired‐end (PE) sequence reads in each genomic library are trimmed; resultant reads are mapped to all reference genomes; binary sequence alignment/map (BAM) files for each sample are combined; and PCR duplicates are marked. The optimum reference genome is established based on mapping‐rate and read‐coverage averages. In step 2, the mapping quality is assessed, extraneous “contaminating” sequences (originating, for example, from the environment, microbes and/or host species) are removed and BAM files are corrected accordingly. In step 3, using gatk, the corrected BAM files are optionally recalibrated; the base quality score recalibration (BQSR) is applied in two iterations against high‐quality variants; initial variants are called; variant call format (VCF) files are created; and variants are then filtered to retain only those which are statistically significant

The *Escalibur* version 0.3‐beta pipeline is presented schematically in Figure 1. It integrates the processing steps using the workflow language WDL version 1.0 (https://github.com/openwdl/wdl), with the steps executed employing the cromwell Workflow Management System version 5.0 (https://github.com/broadinstitute/cromwell). cromwell scales from stand‐alone servers to highly distributed computing environments, and example configuration files for both stand‐alone and distributed computing environments are provided. The WDL‐based implementation and distribution of the pipeline follow the findable, accessible, interoperable and reusable (FAIR) principles (Wilkinson et al., 2016), but can also be readily modified and expanded as required by the user. This pipeline uses a singularity container (Kurtzer et al., 2017) to equip 16 programs (Table S1). In the following, we focus on the three key steps for the identification of genome‐wide nucleotide variability. Each step can be run in a separate workflow in a sequential manner; step 2 is optional.

### 2.1 Step 1. Read‐filtering and mapping

Raw paired‐end (PE) short‐read DNA sequence data (e.g., Illumina or BGISEQ) in FASTQ format (Cock et al., 2010) are optionally filtered for quality (default Phred cut‐off: 20) and adapter‐trimmed using the program trimmomatic version 0.39 (Bolger et al., 2014). Read‐pairs are then mapped to a well‐defined (preferably chromosome‐contiguous) reference genome sequence using the program Burrows‐Wheeler Aligner (bwa) mem version 0.7.12 (Li & Durbin, 2010), PCR duplicates are marked and the resultant BAM files are merged per sample. If multiple genomes or draft genomes are available for a particular species/taxon, the algorithm identifies the most suited (“optimum”) reference genome based on mapping and read coverage averages, providing a sound basis for the identification of nucleotide variations.

### 2.2 Step 2. Quality assessment, and removal of extraneous (contaminating) sequence reads

The quality statistics of trimmed sequence data are calculated using fastqc version 0.11.9 (https://www.bioinformatics.babraham.ac.uk/projects/fastqc). Mapping statistics of trimmed data from each sequence library are calculated using samtools stats version 1.10 (Li et al., 2009); subsequently, key mapping metrics, such as the total numbers of mapped and unmapped reads, percentage of paired reads and the total number of bases mapped, are summarized for each library. To remove extraneous, contaminating sequence reads from the aligned sequence data for individual samples, reads that map to the reference genome are mapped to a spectrum of user‐selected genome sequences available for other organisms using bwa; for example, in the case of a eukaryotic pathogen, reads might be mapped to prokaryotic microbes and the host species. Finally, customized python scripts are used to identify and remove contaminating sequences following the latter mapping phase.

### 2.3 Step 3. Calibration of data and variant calling

The mapped data are then used to identify single nucleotide polymorphisms (SNPs) at individual positions and insertion/deletion events (indels) in relation to the optimum reference genome sequence using gatk version 4.1.3.0. In brief, base quality scores of “raw,” aligned sequence‐read data are recalibrated twice based on the nucleotide variations recorded. Subsequently, for each sample sequenced, SNP sites and indels are identified using the gatk HaplotypeCaller routine and merged into one variant call format (VCF) file, which lists all variable sites for all samples under investigation using gatk CombineGVCFs and GenotypeGVCFs routines. Raw SNP sites and indels are filtered for quality using the gatk VariantFiltration routine, employing best practice for gatk (https://software.broadinstitute.org/gatk/best‐practices).

Specifically, by default, SNP sites are selected (“called”) based on the following criteria: variant confidence (QD) > 2.0; strand bias (FS) < 60.0; mapping quality (MQ) > 40.0; mapping quality (MQRankSum) > −12.5; and read position‐bias (ReadPosRankSum) > –8.0. High‐quality indels are selected according to the following parameters: QD > 2.0; FS < 200.0; and ReadPosRankSum > −20.0. Then, SNP sites are filtered further using the options “‐‐minQ 30” (variant‐quality) and “‐‐minGQ 40” (genotype‐quality) in vcftools version 0.1.17 (Danecek et al., 2011). Note that all filtering parameters above can be configured.

## 3 RESULTS

To validate the *Escalibur* pipeline, we downloaded the soft‐filtered, uncalibrated variant data set (v20210121) from the *Caenorhabditis elegans* Natural Diversity Resource (CeNDR) (Cook et al., 2017). Raw data for the following strains: CB4856 (NCBI accession nos. SRR3440952, SRR3441150, SRR3441428, SRR3441550, SRR3441658 and SRR3452182) and CB4854 (SRR3452139, SRR3441401 and SRR3441123) were obtained from NCBI. These data sets were run separately through *Escalibur* using the genome assembly of the Bristol N2 strain of *C*. *elegans* (GCA_000002985.3; file GCF_000002985.6_WBcel235_genomic.fna.gz) as a reference. Reads were trimmed and mapped, then assessed for potential contaminating reads originating from bacteria (GCF_001484935.1); initial base‐calls remained uncalibrated in the first run and were calibrated in the second run, before variants were definitively called. Variants were filtered (QUAL > 30 && INFO/QD > 20 && INFO/SOR < 5 && INFO/FS < 100 && MIN(FMT/DP) > 5), in accordance with the soft‐filtering approach applied to data in CeNDR, and only homozygous variants were preserved.

The resultant filtered homozygous variants were very similar to those in the CeNDR database (Table 1). The numbers of SNPs called in *Escalibur* for CB4854 (*n* = 80,207–80,228) and CB4856 (*n* = 210,552–211,244) were lower than those in the CeNDR database (i.e., 90,032 and 214,914, respectively). Uncalibrated data had more common SNPs compared with calibrated data for both CB4854 (*n* = 74,411 vs. 74,344) and CB4856 (*n* = 189,597 vs. 187,784). Consequently, smaller numbers of unique SNPs were recorded in uncalibrated than in calibrated data from CeNDR—that is, CB4854 (*n* = 15,621 vs. 15,688) and CB4856 (*n* = 25,317 vs. 27,130)—and using *Escalibur*—that is, CB4854 (*n* = 5,796 vs. 5,884) and CB4856 (*n* = 21,647 vs. 22,768).

**TABLE 1:** Homozygous variants called in data representing strains CB4854^a^ and CB4856^b^ of *Caenorhabditis elegans* using *Escalibur* compared with variants recorded in the CeNDR database. Variants were called separately using calibrated and uncalibrated data

Description	All SNPs (count)	Common SNPs (count; %)	Unique SNPs (count; %)	All indels (count)	Common indels (count; %)	Unique indels (count; %)
CB4854/uncalibrated data						
CeNDR	90,032	74,411; 82.6	15,621; 17.4	42,261	23,803; 56.3	18,458; 43.7
*Escalibur*	80,207	74,411; 92.8	5,796; 7.2	26,081	23,803; 91.3	2,278; 8.7
CB4856/uncalibrated data						
CeNDR	214,914	189,597; 88.2	25,317; 11.8	95,513	59,628; 62.4	35,885; 37.6
*Escalibur*	211,244	189,597; 89.8	21,647; 10.2	68,982	59,628; 86.4	9,354; 13.6
CB4854/calibrated data						
CeNDR	90,032	74,344; 82.6	15,688; 17.4	42,261	23,766; 56.2	18,495; 43.8
*Escalibur*	80,228	74,344; 92.7	5,884; 7.3	26,076	23,766; 91.1	2,310; 8.9
CB4856/calibrated data						
CeNDR	214,914	187,784; 87.4	27,130; 12.6	95,513	58,554; 61.3	36,959; 38.7
*Escalibur*	210,552	187,784; 89.2	22,768; 10.8	68,216	58,554; 85.8	9,662; 14.2
Effect of calibrated vs. uncalibrated data on variants called for CB4854						
Uncalibrated	80,207	79,791; 99.5	416; 0.5	26,081	25,913; 99.4	168; 0.6
Calibrated	80,228	79,791; 99.5	437; 0.5	26,076	25,913; 99.4	163; 0.5
Effect of calibrated vs. uncalibrated data on variants called for CB4856						
Uncalibrated	211,244	207,472; 98.2	3,772; 1.8	68,982	67,259; 97.5	1,723; 2.5
Calibrated	210,552	207,472; 98.5	3,080; 1.5	68,216	67,259; 98.6	957; 1.4

^a^ NCBI accession identifiers PRJNA318647 and SAMN04902526.

^b^ NCBI accession identifiers PRJNA318647 and SAMN04902368.

Similar results were achieved for indels, for which the counts called using *Escalibur* for data relating to strains CB4854 (*n* = 26,076–26,081) and CB4856 (*n* = 68,216–68,982) were lower than those obtained from CeNDR (i.e., 42,261 and 95,513, respectively). Comparing *Escalibur* with CeNDR, more common indels were recorded in uncalibrated than in calibrated data for strains CB4854 (*n* = 23,803 vs. 23,766) and CB4856 (*n* = 59,628 vs. 58,554), whereas fewer unique indels were identified in uncalibrated than in calibrated data: CB4854 (*n* = 15,621 vs. 15,688) and CB4856 (*n* = 35,885 vs. 36,959) from CeNDR, and CB4854 (*n* = 2,278 vs. 2,310) and CB4856 (*n* = 9,354 vs. 9,662) using *Escalibur*. Consistently, more SNPs and indels were shared between resultant *Escalibur* and CeNDR data using uncalibrated rather than calibrated data sets—a result that was expected due to the lack of a calibration step for CeNDR data. When the effect of using calibrated vs. uncalibrated data was assessed, there was no marked increase or decrease in the numbers of variants called (Table 1), with small percentage differences (usually ~0.5% to 2.5%) in the SNPs and indels called.

Calendar time, CPU time and maximum RAM usage for step 1 (1 h 8 min; 36 h 31 m; 31.4 GB), step 2 (39 min; 7 h 45 min; 21.5 GB), step 3, without base call calibration (2 h 38 min; 26 h 31 min; 11.5 GB) and step 3 with base‐call calibration (6 h 25 min; 53 h 50 min; 11.7 GB) were measured using a stand‐alone Linux server, equipped with 48 processing cores and 512 GB of RAM, when running data for both strains (i.e., CB4854 and CB4856) together (Table S2). Calendar time varied, depending on the allocation of available processing cores to programs in configuration files and on generic load of the server during the runs.

## 4 DISCUSSION

Exploring genetic variation within species of nonmodel organisms, such as disease‐causing pathogens, can have important implications, for instance, for understanding the responses of a population to selection pressures, such as environmental changes and drug treatment, and can be central to elucidating pathogen ecology and epidemiology. The use of advanced informatics is enabling population genetic analyses of DNA sequence data sets produced using the latest DNA sequencing technologies. However, the processing of large genomic data sets for “lesser‐studied” organisms has been challenging, and automated pipelines for the rapid analysis of such data sets are scant.

*Escalibur* uses proven cromwell pipeline execution software that allows its integration into a range of computing environments, is reliable and scalable, and supports call‐caching. Therefore, the pipeline can resolve critical and computationally demanding, mapping and calibration steps for SNP‐calling in an automated manner. Moreover, the use of the WDL workflow language allows end‐users to expand and modify the pipeline to suit their needs, as required. Currently, WDL can also be used with execution engines other than cromwell, such as miniwdl (https://github.com/chanzuckerberg/miniwdl; October 13, 2021) and toil (Vivian et al., 2017). However, this workflow has not been tested on these engines. The use of a container to run software packages spares the end‐user from deploying the required tools and their dependencies, which would, otherwise, be a daunting task for researchers without extensive expertise in bioinformatics. Regardless, a familiarity with WDL would be beneficial to addressing issues if they were to arise when running the pipeline.

We limited *Escalibur* to the gatk variant caller due to gatk’s extensive use in the community and its consistent and versatile applicability to data produced on different sequencing platforms (Chen et al., 2019). Although other pipelines for “variant calling” are available, none of them includes key features required for SNP detection in genomes of nonmodel organisms. The automatic selection of a suitable reference genome considers the similarity to genomes inferred for individual samples being studied; if this step is not undertaken, systematic biases or errors may be introduced (Bertels et al., 2014). For instance, the nature and extent of the mapping of sequence reads could vary markedly, depending on the quality of the reference genome assemblies (which could represent population variants of the same species) and/or the coverage of mapped reads to such genomes. Selecting the most “suitable” reference genome sequence prevents SNP errors from occurring and reduces or eliminates uncertainty regarding the identity and relationships of samples under investigation. Recalibration is an essential step to detect systematic errors in base calling by sequencers. To avoid false negatives, due to a lack of defined variants, the pipeline treats highly confident variant calls as genuine.

In this study, the percentage of SNPs (~90%) shared between SNPs available in CeNDR and called using *Escalibur* indicates good performance of the *Escalibur* pipeline. The remaining discrepancy of ~10% is probably due to differences in the methods used in the *Escalibur* pipeline compared with those applied to data deposited in CeNDR. Differences include an additional conversion step of heterozygous to homozygous SNPs in CeNDR, and distinctions in accepted trimming quality and read length. *Escalibur* requires a minimum read length of 40 bp following trimming and a Phred quality of 20 (cf. 20 bp and 15 employed for CeNDR data, respectively). This approach used in *Escalibur* yields more stringent results and somewhat reduced numbers of SNPs and indels compared with data in CeNDR. Calibration has a clear effect on the variants called (≤2.5% variation for strain CB4856), justifying its use. Clearly, *Escalibur* is markedly more stringent in predicting indels than the methods used to create the CeNDR database. Nonetheless, the majority (~90%) of indels were shared between data obtained via *Escalibur* and CeNDR data. The stringent approach used in *Escalibur* and the positive effect of data calibration on variant calling improve the quality of the results and conclusions overall.

Here, our goal was to integrate into *Escalibur* functionalities, such as the selection of the best reference genome and the removal of bias or errors caused by potential contaminating sequences, which are not included in available pipelines. The pipeline does not aim to remove contamination from reference genome sequences, rather only from mapped read data, using user‐selected genomes of organisms or microorganisms that might be possible contaminants. The selection of reference genomes for potential contaminants is usually based on prior knowledge of typical contaminants, or an exploration of the sequence data obtained using programs such as centrifuge (Kim et al., 2016). Our purpose here was to increase functionality, and not to compare the performance of our workflow with that of other published pipelines, as it is well recognized that differing experimental conditions (e.g., quality and preparation of genomic DNA) and procedures (type of library construction and sequencing method) (Chen et al., 2019; Pei et al., 2021) can influence analyses, depending on the workflow system, and thus render the direct comparative evaluation of results challenging or invalid. The present study demonstrates the functionality and the positive effect of data calibration on results overall.

Currently, we are using this workflow routinely for population genomic studies of species of metazoan parasites. With the integration of additional functions (including selection of an optimum reference genome and removal of sequence contaminants), this pipeline should accelerate future phylogenomic and population genetic investigations of eukaryotic pathogens and, potentially, the evaluation of the emergence (evolution) of drug resistance in such populations, and could assist in guiding new strategies to control parasitic and other infectious diseases worldwide. Using the *Escalibur* pipeline, researchers are now able to explore large genomic data sets to gain deep insights into the genetic compositions of, and variation within, populations in just weeks rather than months. Although we established and now employ this pipeline for the analysis of eukaryotic parasites, it has broad applicability to any species.

## CONFLICT OF INTEREST

The authors have declared no conflict of interest.

## AUTHOR CONTRIBUTIONS

The research was planned by P.K.K., N.D.Y., D.W., L.K. and R.B.G., authors B.S., N.G.F., D.W., L.K. and P.K.K. wrote the code to the pipeline, and R.B.G. and P.K.K. wrote the article with input from all other authors.

## BENEFIT‐SHARING STATEMENT

Benefits Generated: Benefits from this research accrue from the sharing of our population genetics pipeline as described above.

## Supporting information

Table S1‐S2

## Data Availability

The pipeline is available at https://gitlab.unimelb.edu.au/bioscience/escalibur (version 0.3‐beta).
